# Miniature Electrochemical Sensing Accelerates Detection of Toxic Responses Induced by Nanoplastics

**DOI:** 10.1021/acsestwater.4c01109

**Published:** 2024-11-22

**Authors:** Haifeng Zhou, Martina G. Vijver, Willie J. G. M. Peijnenburg

**Affiliations:** † Institute of Environmental Sciences (CML), Leiden University, P.O. Box 9518, Leiden 2300 RA, The Netherlands; ‡ Jiangsu Key Laboratory of Advanced Catalytic Materials and Technology, School of Petrochemical Engineering, Changzhou University, Changzhou 213164, P. R. China; § National Institute of Public Health and the Environment (RIVM), P.O. Box 1, Bilthoven 3720 BA, The Netherlands

Emissions of plastic waste will reach ∼53 million metric t per year by 2030. Large pieces of plastics gradually decompose via photodegradation, oxidation, hydrolysis, and mechanical crushing to generate microplastics (MPLs, <5 mm) and nanoplastics (NPLs, <1 μm).^1^ These miniscule particles of plastic are ubiquitous within every compartment: rivers, oceans, soil, and the atmosphere. In addition to the size, composition, and morphology of MPLs and NPLs, toxic substances (such as metal ions, antibiotics, and pesticides) on the plastic surfaces pose a significant threat to both ecological systems and human health. Therefore, it is crucial to understand the dynamics of the plastic-induced toxic response of especially smaller plastic particles (NPLs). Nonetheless, the possibilities for detection of NPLs inducing chronic toxicity and corresponding biomarkers at exposure levels are limited and hinder in-depth investigation of the longer-term mechanisms that induce human and environmental toxicity. It is clear that a more thorough evaluation of exposure and effect levels requires multiplexing, signaling synergies, and more sensitive means of detection. In this Viewpoint, we advocate how electrochemical techniques contribute to the real-time monitoring of the toxicological evolution of organisms exposed to NPLs at low levels and for the long term.

## Use of Electrochemical Sensing Techniques in Response Measurements

The miniaturization of electrochemical sensors enhances their portability, facilitating *in situ*, on-site monitoring and reducing the reliance on centralized laboratory infrastructure.^2^ The high specificity of these sensors, achieved through the incorporation of recognition elements such as enzymes, antibodies, or molecularly imprinted polymers, ensures accurate detection even in the presence of different media.^3^ This sensitivity, combined with rapid response times, allows for real-time or near-real-time analysis, crucial for dynamic systems that require timely data. Additionally, multiple functionalization and structural design strategies for the electrode surface can effectively resist the effects of interference in the monitoring environment and provide a basis for long-term working. The effectiveness of these miniaturized sensors has proven to be highly sensitive and to allow a selective response to potential biomarkers of toxicity such as d-cysteine^2^ and ATP^4^ expressed by cell damage and exposed NPLs.^5^ The integration of miniaturized electrochemical sensors with microfluidic platforms opens up new possibilities for multiplexed, automated analysis in diverse fields. Thus, electrochemical sensors, with their many advantages, offer a more accessible and scalable alternative to traditional methods such as chromatographic or spectroscopic techniques.

## Pervasiveness of the Ecotoxicity of Nanoplastics

**Figure 1:**
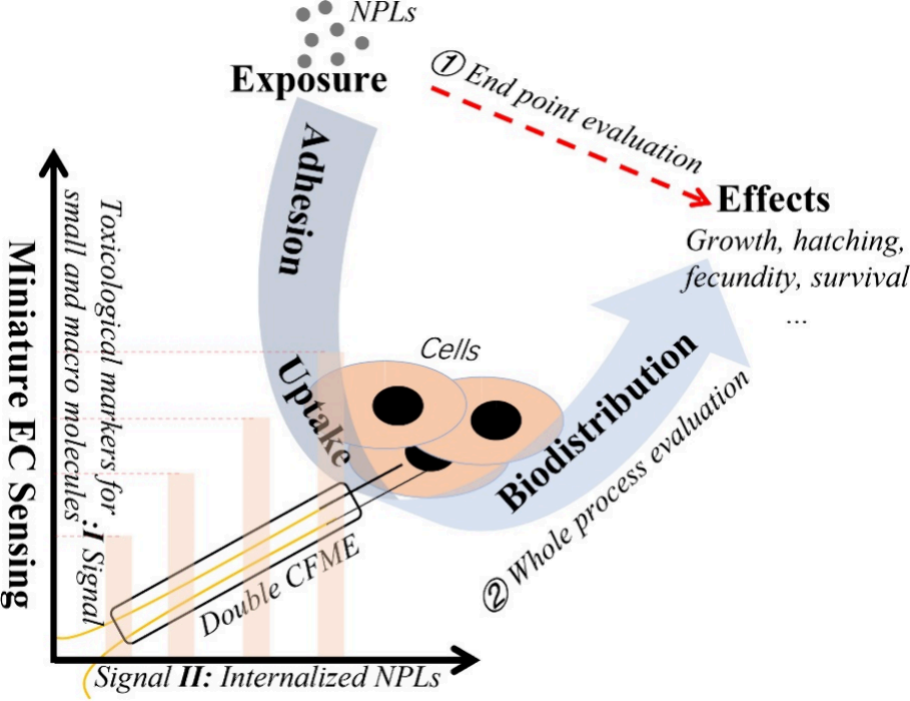
Schematic illustration of the processes of investigating the toxic effect of NPLs. Route ① is the evaluation of ecotoxicity after incubation for a certain period at given concentrations (end point of evaluation). Route ② is monitoring of the toxicological information on NPLs from exposure to effects by electrochemical techniques (whole process evaluation).

NPLs that are smaller, have stronger surface reactivity, and are more mobile are suspected to result in higher risks to organisms than MPLs. Recent research has detected NPLs in aquatic and terrestrial biota, plants, and even human blood, breast milk, lung tissues, and placentas.^6^ Importantly, NPLs often co-occur with other environmental pollutants such as phthalates, metals, antibiotics, pesticides, and persistent organic pollutants (POPs), and this also interferes with the toxic responses. Once NPLs are taken up, the organism usually triggers the overproduction of reactive oxygen species (ROS), which disrupt redox homeostasis, inhibit enzyme activities, and even lead to apoptosis of tissue cells.^7^ Existing methods of toxicological evaluation, specifically based on different *in vitro* or *in vivo* models, have focused predominantly on relatively high exposure concentrations and short-term acute exposures and do not capture the accumulation or effect dynamics (as shown in Figure 1, route 1). Therefore, subtleties of long-term, low-dose exposures and toxicological processes that occur over time have failed to be captured.

## Dynamics Require Real-Time Monitoring in Exposure and Chronic Effects

When organisms are chronically exposed to low levels of NPLs, the formation of less toxic aggregates of a single NPL with tissue or cell surface secretions or with organic matter in the medium is important. With changes in exposure media, aggregates can release a single NPL again and cause effects.^8^ Over time, increasingly subtle impacts emerge, ranging from molecular and cellular changes to alterations at the tissue level, affecting organisms and ecosystems at every level. More importantly, this process embeds essential toxicological information on dose–response relationships, biomarkers, toxicity mechanisms, accumulation dynamics, system susceptibility, and potential ecological impacts, offering a comprehensive framework for assessing the risk of NPLs. If one accounts for these dynamics, there is an urgent need for a method to have real-time monitoring of specific biomarkers in combination with changes in the size and concentration of NPLs over time during exposure, adsorption, *in situ* specifically acknowledging uptake, and biodistribution, and occurrence of adverse effects (e.g., Figure 1, route 2).^8^ The electrochemical sensor can have customizable modification of recognition functional molecules and functional membranes with high selectivity and resistance to interference, which endowed the designed miniaturized electrochemical sensors with the ability to monitor the whole process of toxicity reactions in a continuous manner. With NPL-induced chronic toxicity, cells usually release biomarkers indicative of cellular damage (e.g., causing changes in cell morphology and metabolic disorders), such as production of ROS (e.g., H_2_O_2_ and •OH), RNS (e.g., NO• and ONOO^–^) and generation of endogenous hydrogen sulfide (H_2_S), adenosine triphosphate (ATP), damaged DNA, etc., which are mostly electroactive or highly recognizable (such as the signal I channel in Figure 1). To further determine the cause of the release of different types and amounts of biomarkers, it is important to gain insight into the complete process of gradual internalization of NPLs from extracellular to intracellular (Figure 1, signal II channel), so that mechanistic pathways can be generated. Similarly, on the basis of this multiplexed, mechanistically coupled monitoring strategy, NPLs adsorbed with other environmental contaminants also hold promise for the assessment of their compound toxicity or longer-term toxicity. These dynamic biochemical reaction information, if captured in real time with *in situ* miniature electrochemical sensing and matched to the concentration of internalized NPLs, provides new horizons for toxicological analysis.

## Outlook

We advocate the construction of a synergistic approach to establish new analytical strategies for the multidisciplinary intersection of multiple sensing modalities, facilitating the dynamics of NPL exposure, uptake pathway, and effects. Miniscule novel electrochemical devices ultimately allow for simultaneous *in situ* monitoring of NPLs and of toxicological markers with high spatial and temporal resolution to fill the major knowledge gaps about the mechanisms and evolutionary processes of the toxicity of NPLs to biota (Figure 1, signals I and II).

The signal synergy and high resolution of miniaturized electrochemical sensing allow the following three key aspects of NPL toxicology assessment to be addressed. (1) The amount of NPLs entering the cell is instantly investigated. (2) The alteration of the corresponding cellular state is assessed, e.g., by quantifying changes in the concentration of the biomarkers of the toxic response. (3) The ecotoxicological results induced by plastics that exhibited the cellular population are quantified. Through an integrated toxicological assessment framework that accounts for the effects induced by NPLs that accumulated in organisms and by coexisting pollutant composites, we will be able to effectively capture the complex and dynamic interactions *in situ* between novel environmental pollutant NPLs and human and environmental health, with prospects for real-time measurements.
